# Permanganate of Potassa

**Published:** 1871-03

**Authors:** 


Permanganate of Potassa.—Prof. B. H. Rand tells us, in the *Medical Times*, that we are constantly laboring under a delusion when we use this drug Next to chromic or chloro chromic acid, it is the most active oxidizing agent known; in contact with organic matters it is decomposed and becomes black oxide of manganese and caustic potassa, oxygen being given off. When it is administered internally, the dose is not usually over half a grain, and this is decomposed by two and one-half grains of organic matters. Then, considering the organic contents of the stomach, it must be impossible for any of the permanganate to ever enter the circulation, there to give up its oxygen. The caustic potassa and black oxide of manganese may do the patients good, but this is less probably than is supposed to be the case.

In surgical dressing it should not be applied to the bandages, or by a sponge, as it is at once decomposed by the organic matter of these materials, and the effect in disinfecting the wound, an office which it performs most admirably, is not secured.

The great facility with which organic matter decomposes this salt is shown by its change in impure water, a solution so weak that only a faint pink tinge is perceived losing its color on addition to water containing the slightest organic impurity.

